# Preliminary Comparative Effects of Close‐to‐Nature and Structure‐Based Forest Management on Carbon Sequestration in *Pinus tabuliformis* Plantations of the Loess Plateau, China

**DOI:** 10.1002/ece3.71809

**Published:** 2025-07-13

**Authors:** Jie Zhang, Huiyuan Yang, Youding Zeng, Sakib Al Hassan, Mahmuda Akter Jui, Li Gu

**Affiliations:** ^1^ College of Forestry Northwest A&F University Yangling Shaanxi China

**Keywords:** carbon sequestration, close‐to‐nature forest management, growth partitioning, *Pinus tabuliformis*, structure‐based forest management

## Abstract

*Pinus tabuliformis* plantations on the Loess Plateau face challenges such as poor quality and high mortality rates due to high initial value density and improper thinning practices. To prevent further deterioration of these forests, it is essential to identify suitable forest management methods as soon as possible. Within *Pinus tabuliformis* plantations under different management methods (structure‐based forest management [SBFM], close‐to‐nature forest management [CNFM], and unmanaged), after 5 years of investigation, we analyzed the changes in forest structural complexity and growth partitioning using size inequality (Gini), size–growth relationship (SGR), and growth dominance coefficient (GDC). A linear mixed‐effects model was applied to evaluate the impact of these practices on forest stands. We also compared the trends of the average annual breast height area increment (BAI) and projected the net‐zero timeline after thinning. The results showed that: (1) thinning management temporarily reduced the Gini due to the removal of a certain number of trees. However, the Gini rebounded significantly, and the forest structure became increasingly complex again, and the rebound of SBFM stands was greater than that of CNFM; (2) in the unmanaged stands, larger trees contribute more to stand growth. In the managed stands, the changes in GDC and SGR reflected an increasing contribution of smaller trees to overall growth; and (3) thinning management increased BAI, and this effect became more pronounced over time. Notably, carbon neutrality was projected to be achieved 7.8 years in CNFM stands, which was earlier than the 8.7 years in SBFM stands. These research results will provide a theoretical basis for managing and determining the trees to be harvested for high‐density, low‐quality *Pinus tabuliformis* plantations of the Loess Plateau.

## Graphical abstract

Carbon neutrality was projected to be achieved 7.8 years in stands under close‐to‐nature forest management (CNFM), which was earlier than the 8.7 years in stands under structure‐based forest management (SBFM). CNFM's low‐disturbance characteristics are more sustainable in regions prioritizing ecological restoration, while SBFM could serve as a complementary strategy in extreme scenarios such as severe soil degradation or pest outbreaks.

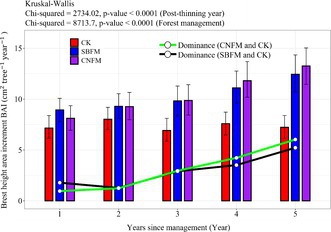

## 1 Introduction

In the context of global climate change, forests are increasingly recognized as critical carbon sequestration systems. However, long‐term unsustainable management practices have resulted in suboptimal productivity across China's plantation forests. To address this, the implementation of thinning strategies has emerged as an essential silvicultural intervention (Eriksson 2006). This practice involves the systematic removal of targeted trees to optimize resource allocation for remaining specimens, thereby enhancing forest regeneration capacity and productive potential (Geng et al. 2021; Zhang et al. 2024).

Forest productivity is fundamentally regulated by three interdependent factors: stand density, structural complexity, and growth partitioning (Forrester 2019). Insufficient stand density directly limits carbon stocks, while underutilized resources from low competition constrain biomass accumulation efficiency. Conversely, excessive density triggers premature self‐thinning processes, leading to premature mortality of suppressed individuals (Pretzsch et al. 2024). Structural complexity manifests through tree size hierarchies resulting from differential resource acquisition efficiency and drives asymmetric competition, particularly for light resources (Lee et al. 2024). During stand development, canopy stratification establishes a competitive hierarchy where dominant trees secure ecological advantage through vertical growth and crown expansion (Binkley et al. 2013; Li et al. 2024). However, there are situations where the growth of large trees may be inhibited, particularly in gusty or arid climates (King 1990; Pretzsch et al. 2022). Such uneven biomass allocations amplify size inequality (Binkley 2004), while changes in tree size‐growth relationships, in turn, impact stand structure (Haemaelaeinen et al. 2024), leading to changes in overall forest productivity.

Contemporary forest management employs targeted silvicultural interventions to achieve multifunctional objectives. Such implements include altering the stand's canopy structure and enhancing the light conditions beneath the forest (Alveshere et al. 2024), reducing the size inequality of trees to adjust forest structure (Liang et al. 2023), or promoting an increase in species diversity and ecosystem diversity (Silva‐Gonzalez et al. 2024; Ming et al. 2020). While managed stands exhibit reduced initial carbon stocks compared to unmanaged counterparts (Sun et al. 2018), they demonstrate accelerated carbon sequestration rates until competitive balance re‐establishes. Management outcomes vary depending on the selection criteria used. Close‐to‐nature forest management (CNFM) and structure‐based forest management (SBFM) are two popular approaches to forest management in China, with widely varying results. Close‐to‐Nature Forestry Theory was first proposed by German forester Karl Gayer in 1898. By developing a structure similar to a natural forest, CNFM integrates the timber production and ecological service functions while maintaining a relatively small spatial scale (such as stand level) (O'Hara 2016). This approach enhances the dynamic balance of forest ecosystems and biodiversity, restoring the natural species and ecological functions (Sujii et al. 2017; Petrokas et al. 2024). There are studies demonstrating its capacity to enhance planted forests' quality and ecological benefits (Sačkov et al. 2017; Wu et al. 2013). In contrast, SBFM was officially proposed by Hui from China in 2007. It is based on three spatial structural parameters—angular measure, degree of mixing, and size class ratio—to quantify forest structure (Wan et al. 2019). These parameters encompass the degree of species mixing within and between species, differences in tree size, and spatial distribution patterns (Hui et al. 2018). This mode mainly structurally regulates the dominant tree species to maintain the forest's overall health (Wan and He 2020).

*Pinus tabuliformis* serves as the primary species used for afforestation on the Loess Plateau due to its exceptional ecological adaptations, including high‐water use efficiency, adaptability to semi‐arid environments, soil amelioration capacity, and effectiveness in preventing soil erosion (Zhang et al. 2015). However, excessively high initial planting density of *Pinus tabuliformis* plantations in this region, along with a lack of timely nurturing, has led to significant natural thinning. As a result, tree mortality rates are increasing annually. This trend negatively impacts the expected results of the artificial forests and causes unnecessary wastage of forest resources (Li and Wang 2009), and highlights the critical need for developing scientific forest management modes tailored to *Pinus tabuliformis* plantations.

We assumed that the dynamic changes in size inequality, growth partitioning, and above‐ground carbon stock of *Pinus tabuliformis* plantations under CNFM and SBFM would differ significantly. We analyzed the patterns of these changes and their interrelationships to uncover how different forest management methods enhance carbon sequestration in this kind of forest. The findings will establish a theoretical foundation for managing *Pinus tabuliformis* plantations on the Loess Plateau and guide decisions about wood harvesting and retention based on diverse management objectives.

## 2 Data and Methods

### 2.1 Study Area

**FIGURE 1:**
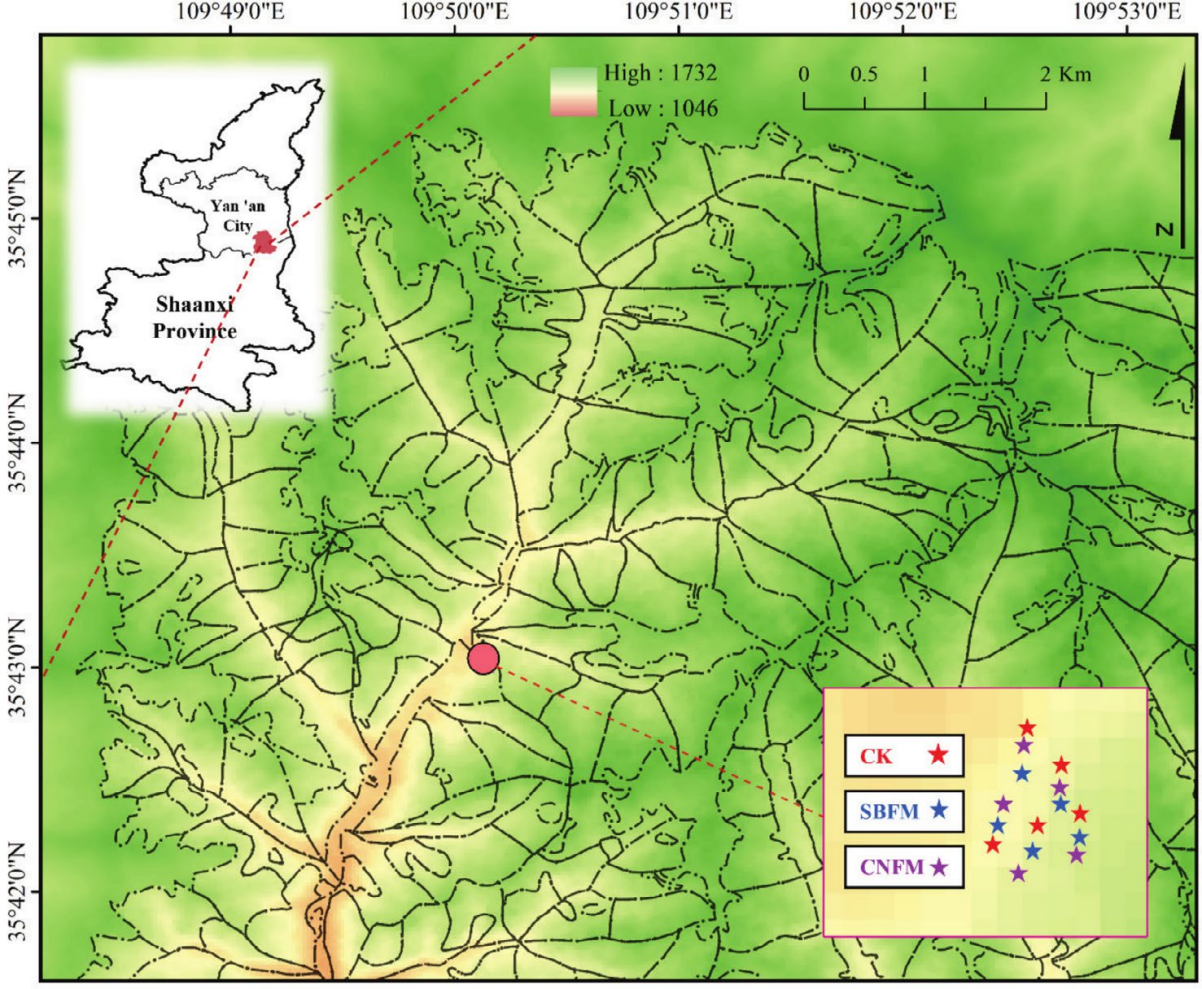
The study sites are in the Huanglong Mountain area of Shaanxi Province, China. The plots are marked by five‐pointed stars, with different colors representing various management methods. CK, unmanaged stands; CNFM, close‐to‐nature forest management stands; SBFM, structure‐based forest management stands.

The study area is located in the Huanglong Mountain region (35°28′46′′–36°02′01′′ N, 109°38′49′′–110°12′47′′ E), southeast of the Loess Plateau in northern Shaanxi (Figure 1). The climate in this area is monsoon continental montane climate; the landform belongs to the loess landform type with ravines and gullies; the average annual temperature is 7.6°C–10.2°C; the annual precipitation is about 600 mm; the altitude of the study area is between 1100 and 1500 m. The Huanglong Mountain area has a great variety of plant species, with dominant species mainly including Chinese Pine ( *Pinus tabuliformis* ), lacebark pine ( *Pinus bungeana* ), elm ( *Ulmus pumila* ), Chinese wingnut ( *Pterocarya stenoptera* ), and mono maple (*Acer pictum* subsp. *mono*). The area of *Pinus tabuliformis* reaches 54.08 km^2^, accounting for 31.77% of the total forest area.

### 2.2 Study Site and Data Collection

In 2017, based on in situ trekking, we established fifteen 20 × 30 m plots. During the 2018 implementation phase, these plots were assigned to three experimental groups (five plots each): structure‐based forest management (SBFM), close‐to‐nature forest management (CNFM), and unmanaged control. The harvesting intensity for the managed stands ranged from 26.2% to 30.9% (based on volume), because moderate‐intensity thinning can enhance the growth of *Pinus tabuliformis* plantations, and high‐intensity thinning (such as removing more than 40% of the forest volume) will disrupt the original spatial heterogeneity of the forest stand, causing the distribution of trees to tend towards uniformity (Li et al. 2014). Buffers were established around all stands to mitigate the effects of forest edges and adjacent treatments. Except for the different forest management methods, these plots had highly similar environmental conditions.

Under CNFM, we designated *Pinus tabuliformis* as the target tree species and promoted natural regeneration through protecting seed dispersal and germination processes. Our overall stand management approach leans towards continuous cover forestry. We carried out selective harvesting operations. The stand density was regulated by removing competing trees that impede the growth of target trees, underperforming individuals, and deadwood, while retaining tree species with high local economic value, such as oaks and poplars.

Under SBFM, we retained valuable, rare, and endangered species, and removed poorly growing trees affected by pests and diseases, or that had hollow or decayed trunks. We also cleared out clusters of multiple trees and removed competing trees that obstructed the growth of the retained trees to minimize shading and overcrowding. To increase tree species diversity, we prioritized the removal of trees of the same species located on one side of the retained trees.

From 2018 to 2023, we conducted annual surveys, recording basic metrics such as tree diameter at breast height (DBH), height, crown width, and geographic location. Detailed information for each stand is presented in Table 1.

**TABLE 1:** Basic characteristics of plots and intensity of thinning.

Management	Plot	Elevation (m)	Slope (°)	Aspect	Slope position	Age	*N* (trees hm^−1^)	Thinning intensity (by volume) (%)
SBFM	S1	1426	15	North	Middle	34	3343	24.9
	S2	1436	17	North	Middle	34	3543	28.9
	S3	1453	21	North	Upper	34	3643	27.1
	S4	1463	19	North	Upper	34	3420	25.2
	S5	1443	18	North	Middle	34	3730	26.1
CNFM	N1	1463	23	North	Upper	34	3369	23.3
	N2	1423	14	North	Middle	34	3732	25.2
	N3	1453	18	North	Upper	34	3832	30.2
	N4	1443	17	North	Middle	34	3572	27.1
	N5	1446	20	North	Middle	34	3523	26.7
CK	C1	1457	20	North	Middle	34	3633	—
	C2	1463	18	North	Upper	34	3481	—
	C3	1438	22	North	Middle	34	3788	—
	C4	1454	14	North	Upper	34	3379	—
	C5	1442	17	North	Middle	34	3533	—

*Note:* “—” Indicates data not available in the original tables.

Abbreviations: CK, unmanaged stands; CNFM, close‐to‐nature forest management stands; SBFM, structure‐based forest management stands.

### 2.3 Gini Coefficient Calculation

Size inequality was quantified using the Gini coefficient (Britwum et al. 2022), a robust metric introduced by the Italian economist Corrado Gini in 1922, based on the Lorenz curve and reflecting the extent of differences in income distribution (Sun et al. 2010). The Gini coefficient is relatively insensitive to changes in sample size but effectively distinguishes between the differently shaped DBH distributions (Lexerød and Eid 2006). In our study, the coefficient was calculated through bootstrap resampling (*n* = 1000 iterations) with the following implementation (Cordonnier and Kunstler 2015):

(1) $$\text{Gini}=2\frac{\sum_{i=1}^{n}i{\mathrm{DBH}}_{i}}{n\sum_{i=1}^{n}{\mathrm{DBH}}_{i}}-\frac{n+1}{n}$$

where *n* represents the number of trees in each plot, and *i* = (1 … *n*) is the order of the trees; ${\mathrm{DBH}}_{i}$ refers to the diameter at breast height (cm) of trees; Gini ranges from 0 (perfect equality) to 1 (maximum inequality).

### 2.4 Growth Partitioning Variables Calculation

While size inequality effectively quantifies the distribution of differently sized trees within stands, it fails to account for the distribution of stand productivity among trees of varying sizes (Forrester 2019). To address this limitation, we are using the size–growth relationship (SGR) and the growth dominance coefficient (GDC) to quantify the growth distribution increments among forest stands with trees of varying sizes (Baret et al. 2017; Looney and Zhang 2022).

The SGR is a stand‐level metric that indicates the relative efficiency of trees of various sizes in utilizing resources, thereby enhancing our understanding of the factors influencing stand productivity and size heterogeneity (Pretzsch and Biber 2010). It is defined as the slope of the linear regression between the tree growth ratio (the percentage of total stand growth) and the tree size ratio (the percentage of total stand size) (Metsaranta and Lieffers 2010). To calculate SGR, we first transform the ratio of an individual tree breast height area increment to its breast height area ratio using the centered log‐ratio transformation. The transformed growth ratio is then analyzed through simple linear regression against the individual tree's breast height area. If the growth of all trees is proportional to their size, the slope SGR will equal 1, indicating that tree growth is completely symmetrical (the growth amount is proportional to their size). Conversely, if SGR is greater than 1, larger trees are growing disproportionately faster than their diameter; if SGR is less than 1, it suggests that smaller trees are growing more quickly relative to their size. Similar to the Gini coefficient, SGR is calculated using the bootstrap resampling method. Additionally, to further analyze the growth rates of retained trees under different thinning intensities, this method is also applied to calculate the average annual basal area increment (BAI) of all retained trees within the plot.

(2) $$\mathrm{BAI}=\frac{\sum_{i=1}^{n}∆G_{i}}{n}$$

where n represents the number of trees in each plot; $∆G_{i}$ refers to the annual increments of the breast height area at the tree.

Meanwhile, the GDC describes the relative contribution of different DBH classes of trees to the total growth of a forest stand (Binkley 2004). According to the GDC calculation method proposed by West (2014), the breast height areas of trees within a plot are arranged in ascending order. This arrangement allows for calculating the cumulative proportion of each breast height area relative to the total breast height area of the stand. Additionally, it calculates the cumulative proportion of each tree's breast height area increment relative to the total breast height area increment of the stand. The GDC represents the area under the curve of the cumulative relative values between tree breast height area and breast height area growth, indicating the relative contribution of larger or smaller trees to the total growth of the stand. A sample size of 50 trees is sufficient for SGR analysis (Bose et al. 2021). Since GDC involves the relationship between cumulative breast height area and the cumulative growth of breast height area (Lemire et al. 2020), its calculation requires the breast height area and its increment of all trees in each sample plot. The formula is as follows:

(3) $$\mathrm{GDC}=1-\sum_{i=1}^{n}\left(s_{i}-s_{i-1}\right)\left(\Delta_{i}+\Delta_{i-1}\right)$$

where *n* represents the number of trees in each plot and *i* = (1 … *n*) is the order of the trees; $s_{i}$ and $s_{i-1}$ refers to the tree breast height area; $\Delta_{i}$ and $\Delta_{i-1}$ refers to the tree annual increments, which is the breast height area difference of the tree in the given year and the previous year. GDC = 0 indicates the contribution of tree growth to total stand growth is proportion to size; GDC > 0 means that the growth of bigger trees contributes disproportionally more to the stand growth; conversely, GDC < 0 indicates that smaller trees account for a greater contribution to stand growth than their contribution to stand basal area.

Both metrics have been employed in numerous studies to examine changes in stand growth partitioning with age across different tree species and regions (Binkley and Kashian 2015; Britwum et al. 2022), to analyze the effects of species composition and size inequality on ecological processes (Pommerening et al. 2016), or to investigate the impacts of megadroughts on stand growth partitioning and size inequality (Bose et al. 2021). The GDC and SGR provide complementary insights into our studies of stand growth partitioning (Forrester 2019) and serve as useful indicators for assessing the redistribution of stand resources following forest management and for identifying trees that should be removed during management activities (Dye et al. 2019).

### 2.5 Above‐Ground Carbon Stock Calculation

The above‐ground carbon stock of stands can be estimated through biomass. In this study, we used the biomass model for individual *Pinus tabuliformis* proposed by Xiao (1990) specific to the Huanglong Mountain area to estimate the biomass of *Pinus tabuliformis* . We adopted the model Zeng (2017) proposed to estimate other tree species' biomass. The specific models are as follows:

(4) $$W=\sum_{i=1}^{n}0.02112{\left(D^{2}H\right)}^{1.13674}$$

(5) $$W^{\prime }=c\times D^{\frac{7}{3}}$$

where W refers to the biomass (kg) of each tree; $W^{\prime }$ represents the biomass of each tree of other species; D and H are diameter at breast height (cm) and the height of each tree; and c indicates the parameters of biomass model and the specific values are shown in Table 2.

**TABLE 2:** Biomass model parameters and carbon content coefficients by species.

Species	Parameter of biomass model *c*	Coefficient of carbon content $r_{j}$
*Quercus* L.	0.1729	0.4827
*Betula* L.	0.1454	0.4897
Other hardwood species	0.1875	0.4901
Other softwood species	0.1329	0.4502
*Populus* L.	0.1253	—
*Ulmus* L.	0.1374	—
*Pinus tabuliformis*	—	0.5184
Shrub	—	0.4540
Herb	—	0.4520

*Note:* “—” Indicates data not available in the original tables.

According to LY/T 2260‐2014, LY/T 2658‐2016, and LY/T 2659‐2016, we assessed the forest's above‐ground carbon stock in the study area using the carbon coefficients for the above‐ground portions of trees (National Forestry Administration of the People's Republic of China 2014, 2016a, 2016b). Additionally, we used the carbon conversion coefficients for shrubs and herbaceous plants in the *Pinus tabuliformis* artificial forest ecosystem of the Loess Plateau region from research conducted by Yang et al. (2014).

(6) $$W=\sum_{i=1}^{n}W_{i}$$

(7) $$C=r_{j}\times W$$

where n is the number of trees in the stand; W is the biomass of all trees in each plot; $W_{i}$ is the biomass of each tree; C is the forest carbon density (t C ha^−1^); and $r_{j}$ indicates the conversion coefficient of the carbon content rate, and the specific values are shown in Table 2.

### 2.6 Data Analysis

Before conducting the data analysis, we created histograms to visualize the distribution of tree diameters (DBH) for each plot, illustrating the changes in tree diameter before and after management. Our statistical analysis assessed the changes over time in the Gini, GDC, and SGR of the stands, comparing three different management methods. We employed the two‐sample Wilcoxon test to assess whether there were significant differences between various periods for each method. By discussing these metrics alongside the actual changes in average annual breast height area increment (BAI cm^2^ tree^−1^ year^−1^) and the carbon stock, we gain a clearer understanding of the growth conditions of the stand.

To further quantify the effects of management methods on forest size diversity and size‐growth relationships, the “lmer” package in R (version 4.4.2) was used to fit a linear mixed‐effects model. Gini, SGR, and BAI (stand level m^2^ ha^−1^ year^−1^) were set as response variables, while management methods (*M*), years since management (YSM), *M* × YSM, YSM^2^, and *M* × YSM^2^ were used as explanatory variables to fit multiple candidate models. *M* × YSM, YSM^2^, and *M* × YSM^2^ represent potential interactions and possible nonlinear trends over time. There is also natural variation in the starting breast height area (m^2^ ha^−1^ year^−1^) for different plots, leading the statistical model to underestimate the random variation, so we introduce the starting BA as a fixed effect in the BAI model. Considering the difference in response variables among plots at the beginning of the investigation, random effects of plots were included to avoid the dependency resulting from repeated measurements (Zuur et al. 2009). Finally, the performance of the models was compared based on the Akaike Information Criterion (AIC). The complete definitions and units of all acronyms are listed in Table 3.

(8) $$\begin{array}{c} {\text{Response}}_{\mathrm{ij}}=\left(\beta_{0}+u_{0i}\right)+\beta_{1}M+\beta_{2}\mathrm{YSM}\\ +\beta_{3}\left(M\times \mathrm{YSM}\right)+\beta_{4}{\mathrm{YSM}}^{2}+\beta_{5}\left(M\times {\mathrm{YSM}}^{2}\right)+\varepsilon_{\mathrm{ij}} \end{array}$$

where ${\text{Response}}_{\mathrm{ij}}$ is the value of the response variable (Gini, SGR, BAI) at the *j*th survey under ith management method; M represents the forest management method (SBFM, CNFM, and unmanaged); YSM is the number of years since management; $\beta_{0}$ is the mean value of each horizontal intercept of plots; $u_{0i}$ is the difference between each level intercept and the mean intercept; $\beta_{1}–\beta_{5}$ is the estimated parameter of the fixed effects component; and $\varepsilon_{\mathrm{ij}}$ is the model estimation error.

**TABLE 3:** Explanation of acronyms and units used in the study.

Acronym	Full name	Definition
GDC	Growth dominance coefficient	Unitless (range: −1 to 1)
SGR	Size–growth relationship	Unitless (slope)
YSM	Years since management	Year
CNFM	Close‐to‐nature forest management	Treatment category
SBFM	Structure‐based forest management	Treatment category
BAI	Annual breast height area increment	cm^2^ tree^−1^ year^−1^ (tree‐level)
		m^2^ ha^−1^ year^−1^ (stand‐level)

## 3 Results

### 3.1 Stand DBH Distribution Before and After Thinning

**FIGURE 2:**
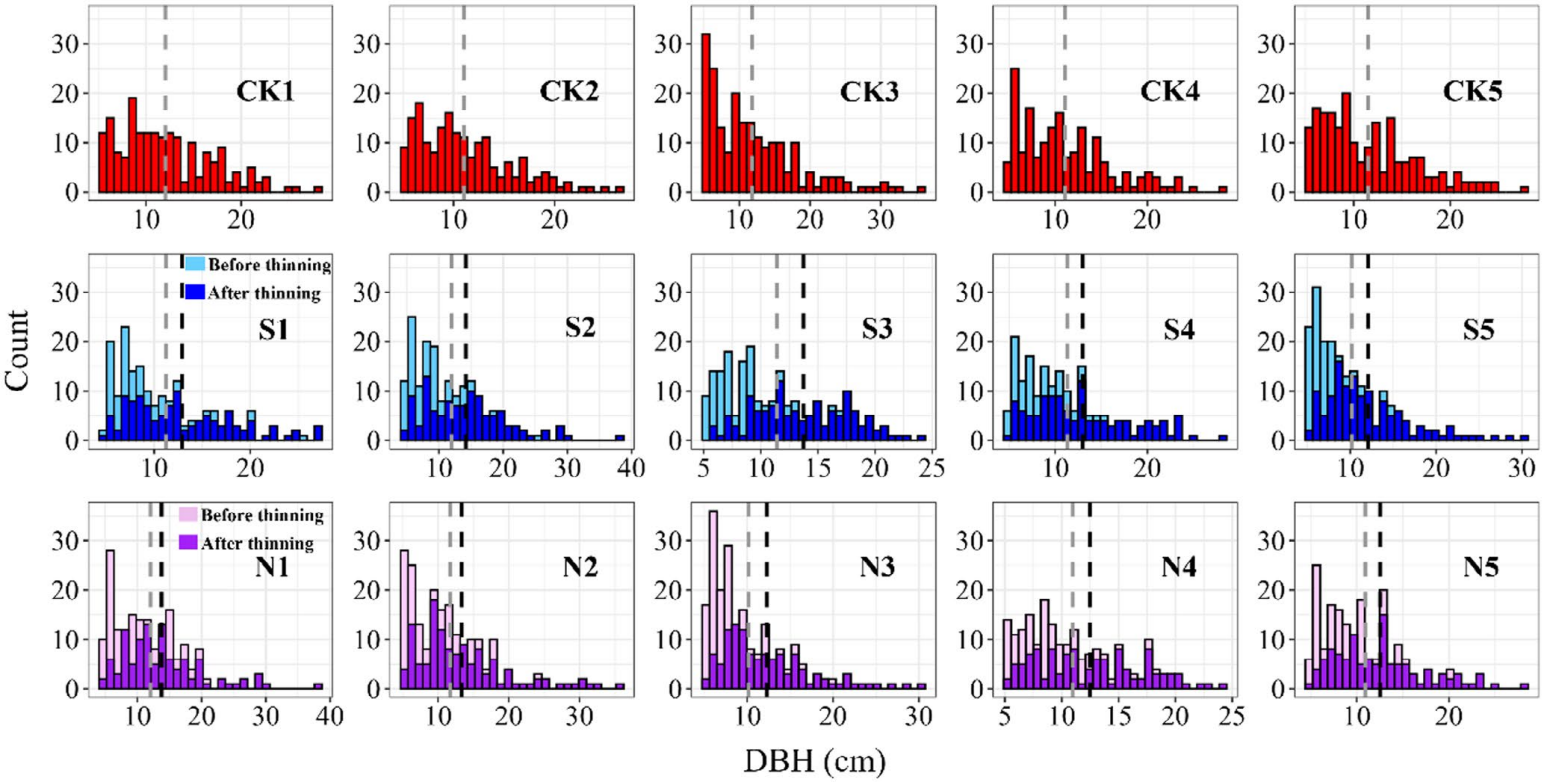
Distribution of DBH in the stands. The red, blue and purple map respectively represents the DBH distribution of the control forest, the SBFM forest, and the CNFM forest (the light color represents the situation before thinning, and the dark color represents the situation after thinning). The vertical dashed line is the average DBH of the stand (gray represents unmanaged, and black represents after management); CK indicates unmanaged stands; S indicates stands with structure‐based forest management (SBFM); N indicates stands with close‐to‐nature forest management (CNFM).

We plotted the DBH distribution graph of trees (DBH > 5 cm) before and after management (Figure 2). The results indicated that, before thinning, most trees concentrated in the DBH range of 5–20 cm, reflecting a clear trend of smaller trees being predominant in the overall stand. After thinning, the vertical line representing the average tree DBH shifted notably to the right, indicating an increase in the average DBH. Table 4 shows that the mean DBH of the control forest was 11.3 ± 4.7 cm. The BFM forest had a mean DBH of 11.2 ± 4.9 cm before harvest, and the mean DBH after thinning increased to 13.0 ± 5.3 cm, with an increase in mean DBH of about 1.8 cm. The CNFM forest, on the other hand, had a mean DBH of 11.1 ± 4.9 cm before harvest, and the mean DBH after thinning increased to 13.4 ± 5.4 cm, an increase in mean DBH of about 2.3 cm. This suggests that trees removed during SBFM had a larger average DBH (8.9 ± 3.3 cm) compared to CNFM (8.7 ± 3.0 cm). Although thinning significantly improved the structure of DBH distribution, the post‐thinning distribution still exhibited a clear right‐skewed characteristic. This indicated that the numerical prevalence of smaller trees has diminished, but it has not entirely vanished.

**TABLE 4:** Diameter characteristics of harvested and residual trees in *Pinus tabuliformis* plantations under different management methods.

Management	Average DBH (cm)		
	Pre‐thinning	Post‐thinning	Harvested
SBFM	11.2 ± 4.9	13.0 ± 5.3	8.9 ± 3.3
CNFM	11.1 ± 4.9	13.4 ± 5.4	8.7 ± 3.0
CK	11.3 ± 4.7	—	—

*Note:* “—” Indicates data not available in the original tables.

Abbreviations: CK, unmanaged stands; CNFM, close‐to‐nature forest management stands; SBFM, structure‐based forest management stands.

### 3.2 Size Inequality Analysis

**FIGURE 3:**
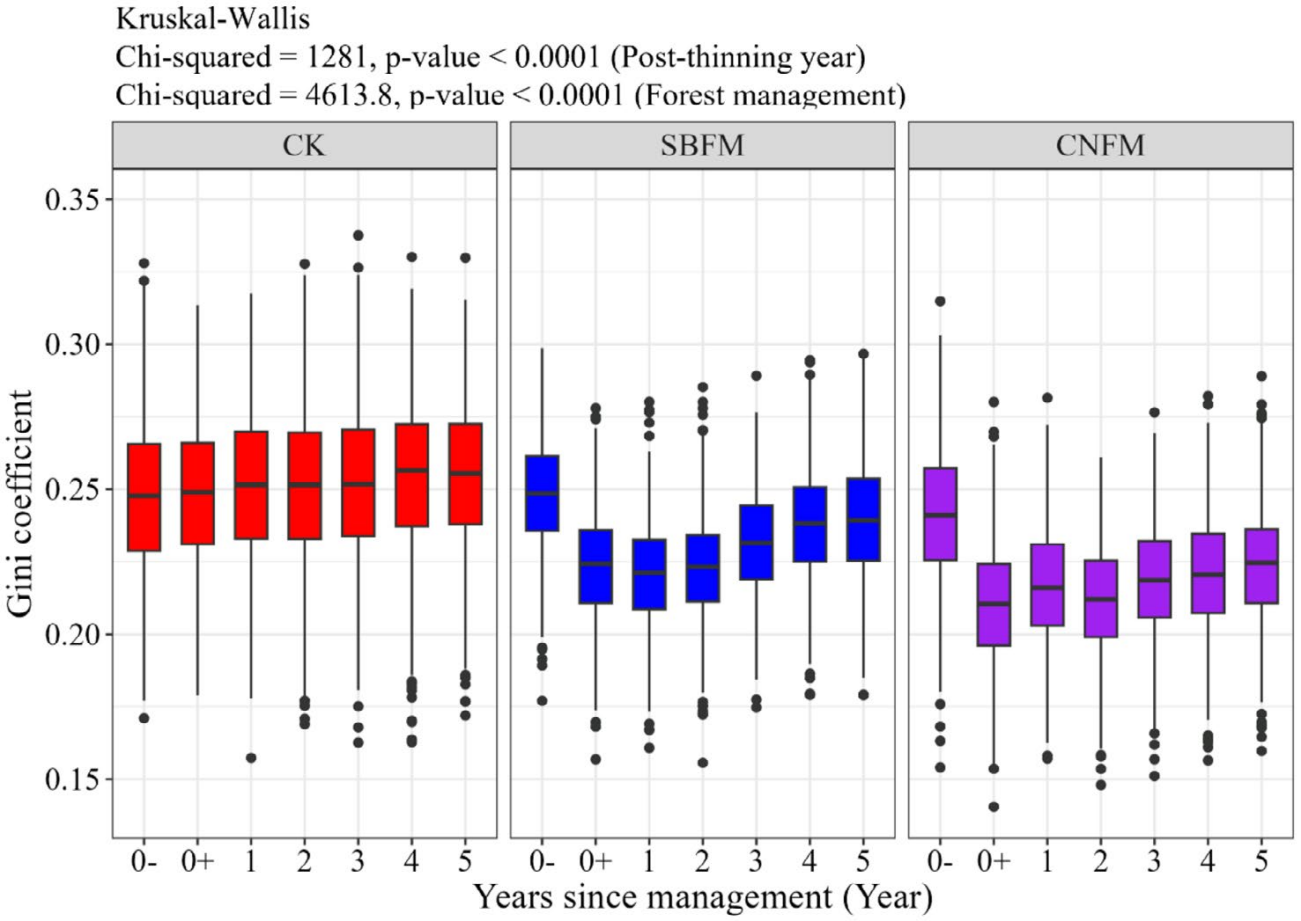
Size inequality (Gini) variation over time under three different management methods. (0−: Before thinning in 2018; 0+: After thinning in 2018). CK, unmanaged stands; CNFM, close‐to‐nature forest management stands; SBFM, structure‐based forest management stands.

The results of the two‐sample Wilcoxon test showed (Figure 3) that the Gini for unmanaged stands increased slowly and steadily over time, and the Gini for managed stands exhibited significant variation and clear trends (*p* < 0.05). Specifically, the Gini for stands under SBFM and CNFM decreased by approximately 0.05 after thinning, followed by a noticeable upward trend. SBFM stands demonstrated a more pronounced Gini rebound compared to CNFM stands. Notably, by the fifth year after thinning, the Gini for managed stands had not yet reached the levels observed before thinning.

### 3.3 Stand Growth Partitioning

**FIGURE 4:**
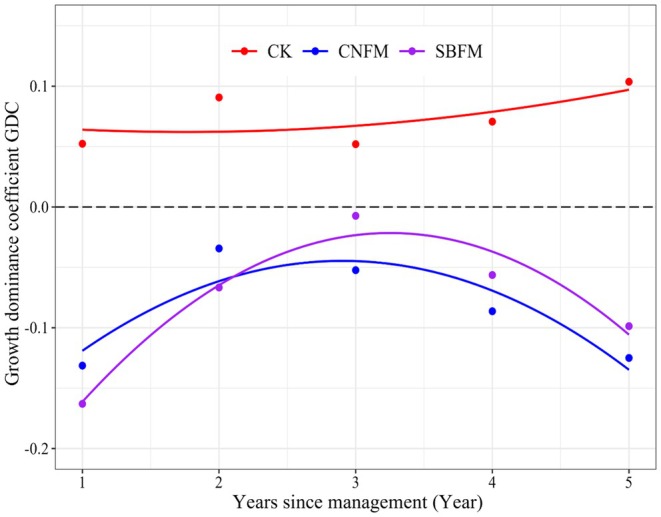
The growth dominance coefficient (GDC) variation over time is under three different management methods. The line with GDC = 0 indicated that the growth of larger (smaller) trees contributed to the total stand growth in proportion to their size, that is, no growth dominance existed. CK, unmanaged stands; CNFM, close‐to‐nature forest management stands; SBFM, structure‐based forest management stands.

The temporal dynamics of growth dominance coefficient (GDC) were fitted using quadratic regression of post‐thinning years (Figure 4). GDC of all stands ranged from −0.2 to 0.15. Among these, the GDC for unmanaged stands remained above 0 throughout the survey period, exhibiting an upward trend. In contrast, the GDC for the managed stands was below 0 in the first year, followed by a trend of rising‐then‐decreasing. Specifically, the GDC for stands under SBFM began to decline after increasing for 2 years, while the GDC for stands under CNFM started to decrease after 1 year of growth.

**FIGURE 5:**
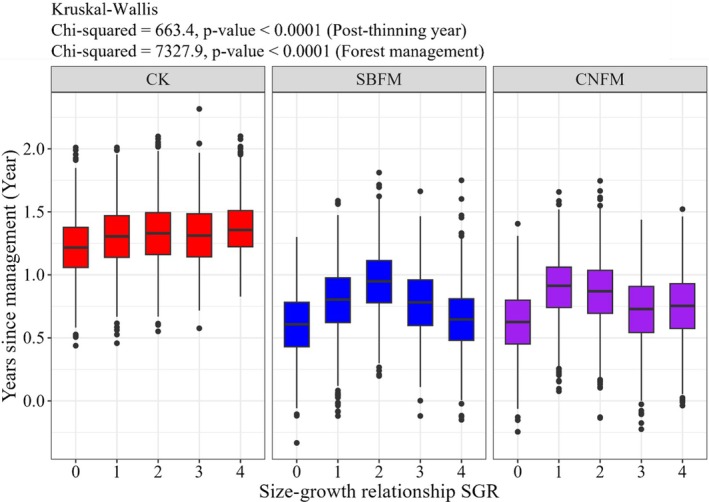
The size–growth relationship (SGR) varies over time under three different management methods. The line with SGR = 1 indicates that the growth of all trees is proportional to their size. CK, unmanaged stands; CNFM, close‐to‐nature forest management stands; SBFM, structure‐based forest management stands.

The results of the two‐sample Wilcoxon test indicated that the differences in SGR across different survey periods for all stands were statistically significant (*p* < 0.05) (Figure 5). The SGR for unmanaged stands remained above 1, showing a continuous upward trend. In contrast, the SGR for managed stands consistently fell below 1, demonstrating an inverse size asymmetry. Specifically, the SGR for managed stands exhibited an upward‐then‐downward trend. The SGR for stands under CNFM began to decline after the first year, while the SGR for stands under SBFM started to decrease after the second year.

### 3.4 Forest Management Methods Effects

The fixed effects of the final model of Gini include *M*, YSM and *M* × YSM, and for the SGR final model included *M*, YSM, and YSM^2^; which of BAI final model included YSM, YSM^2^, and M × YSM (Table 5). The response of size inequality, stand growth partitioning between individual trees and BAI were related to the management mode. These variables significantly impact the dynamic changes of Gini, SGR, and BAI (*p* < 0.05). M showed a significant negative effect on Gini, SGR, and BAI. The main effect of YSM varied from model to model. The YSM showed a positive linear effect in the Gini model, while the BAI model showed a negative linear but positive quadratic effect, and the linear and quadratic terms of YSM in the SGR model together revealed a nonlinear relationship. In addition, the interaction between *M* and YSM was highly significant in Gini and BAI model, indicating that years since management significantly moderated the effect of management mode on the size inequality and BAI. In particular, the BAI model results showed that stands under CNFM exhibited significantly higher BAI (1.105 vs. 0.916 cm^2^ tree^−1^ year^−1^) compared to stands under SBFM, suggesting that single‐tree carbon accumulation trajectories varied with management mode and years since management.

**TABLE 5:** Fixed effect coefficients, standard errors, and *t*‐test results of optimal models.

Model	Explanatory terms	Estimate	SE	*t* Value	*p*
GINI	(Intercept)	0.26748	0.006642	40.273	9.15E‐18***
	CNFM	−0.04686	0.009	−5.20661	0.000139***
	STFM	−0.0447	0.009	−4.96662	0.000215***
	YSM	−0.00962	0.001721	−5.58752	7.05E‐07***
	YSM^2^	0.001943	0.000271	7.156927	1.9E‐09***
	CNFM:YSM	0.00434	0.000787	5.516147	9.18E‐07***
	STFM:YSM	0.0049	0.000787	6.227908	6.46E‐08***
SGR	(Intercept)	0.972313	0.044165	22.01557	2.13E‐20***
	CNFM	−0.22298	0.054869	−4.0639	0.000618***
	STFM	−0.22448	0.054869	−4.09124	0.00058***
	YSM	0.160617	0.019113	8.40375	1.68E‐11***
	YSM^2^	−0.02351	0.003014	−7.79898	1.66E‐10***
	CNFM:YSM	−0.01786	0.008737	−2.04426	0.045641*
BAI	CNFM	−0.80958	0.139938	−5.78529	5.63E‐05***
	STFM	−0.31199	0.137562	−2.26802	0.040341*
	Starting BA	0.093717	0.018562	5.048806	0.000373***
	YSM^2^	0.010667	0.004952	2.154244	0.035539**
	CNFM:YSM	0.261896	0.014351	18.24884	3.08E‐25***
	STFM:YSM	0.219834	0.014351	15.31802	1.11E‐21***

Abbreviations: CK, unmanaged stands; CNFM, close‐to‐nature forest management stands; SBFM, structure‐based forest management stands; Starting BA, starting breast height area; YSM, years since management.

* Represents within statistical significance level 5%.

** Represents within statistical significance level 1%.

*** Represents within statistical significance level 0.1%.

*M* has a significant negative effect on Gini, SGR, and BAI. The main effect of YSM varies from model to model. In the Gini model, YSM exhibited a positive linear effect, whereas in the SGR model, it showed a negative linear effect. Across all three models, the quadratic term of YSM revealed significant nonlinear relationships. Furthermore, the interaction of *M* with YSM is highly significant in the Gini and BAI models, suggesting that the number of years since management significantly moderates the effect of *M* on size inequality and BAI. In particular, results from the BAI model indicated that BAI was significantly higher in CNFM stands (0.262 m^2^ ha^−1^ year^−1^) than in SBFM stands (0.220 m^2^ ha^−1^ year^−1^), suggesting that the forest carbon accumulation trajectory varied with management mode and the number of years since management. As a baseline variable in the BAI model, BA showed a significant positive effect (*β* = 0.094, *p* < 0.001), helping to explain the differences in BAI across management modes.

### 3.5 BAI and Above‐Ground Carbon Stock

**FIGURE 6:**
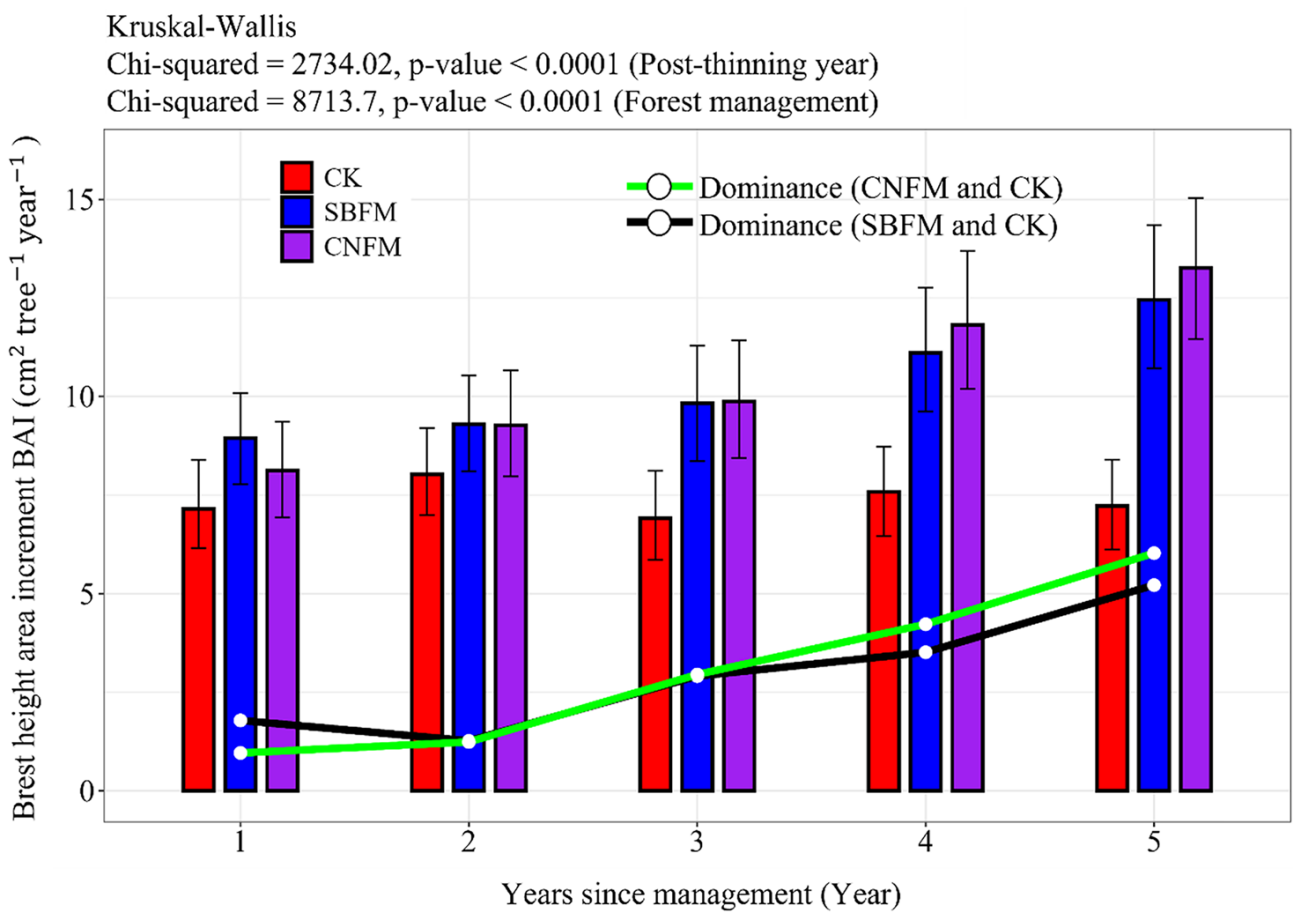
Changes in BAI over time during 5 years after management in stand. CK, unmanaged stands; CNFM, close‐to‐nature forest management stands; SBFM, structure‐based forest management stands.

The BAI of single trees in different years is as follows: CNFM (8.135, 9.237, 9.894, 11.782, 13.287) > SBFM (8.933, 9.314, 9.835, 11.134, 12.464) > CK (7.124, 8.045, 6.923, 7.585, 7.724) (cm^2^ tree^−1^ year^−1^) (Figure 6). Here, the BAI of managed stands showed a continuous increase over time, and a growing difference compared to unmanaged stands. In the early stages of the survey, stands under SBFM had a higher BAI than CNFM. However, by the end of the survey, the BAI of CNFM stands had surpassed that of SBFM stands.

**FIGURE 7:**
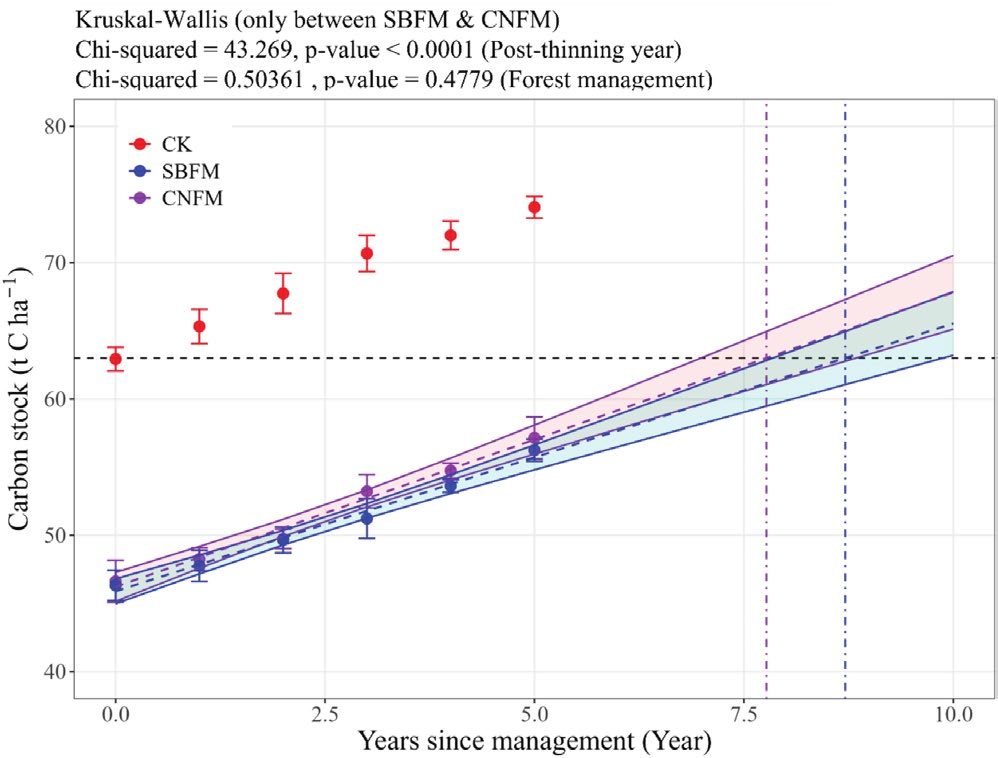
Carbon stock recovery and projected net‐zero timelines after thinning. The gray horizontal line in the middle indicates the baseline average carbon stock (63.0 t C ha^−1^) before thinning in 2018. The two vertical lines on the right are the net zero predicted time markers of the stands under SBFM (8.7) (purple) and CNFM (7.8) (blue).

The carbon stock recovery and projected net‐zero timelines after thinning (Figure 7) demonstrate significant variations in change tendency of the above‐ground carbon storage under different forest management regimes (*p* < 0.05). Specifically, the stands under CNFM resulted in a carbon stock loss of 15.9 t C ha^−1^ during the harvest year, with the stands under SBFM showing a higher depletion of 16.3 t C ha^−1^. Predictive modeling indicates that carbon neutrality of CNFM stands is projected to be achieved in 7.8 years, whereas SBFM stands require a marginally longer recovery period of 8.7 years to reach the net‐zero threshold.

## 4 Discussion

Focusing on size inequality, growth partitioning of different‐sized trees, breast height area increment (BAI), and above‐ground carbon stock, this study quantitatively analyzed the dynamic changes in *Pinus tabuliformis* plantations under SBFM, CNFM, and unmanaged. Managed stands exhibited enhanced resource acquisition capacity in residual smaller trees, with distinct trajectories in size inequality development and growth partitioning patterns between treatments. While SBFM induced greater fluctuations in size inequality and growth repartitioning, CNFM demonstrated superior short‐term growth stimulation.

### 4.1 Dynamic Changes in Size Inequality and Growth Partitioning

In the optimal models, the impact of STFM on both Gini and SGR was greater than that of CNFM, indicating fundamental differences in size diversity and growth partitioning between management regimes. Generally, thinning (typically targeting smaller trees) can reduce the size diversity within the stand (Soares et al. 2017; Zhao et al. 2022). In this experiment, managed stands exhibited more noticeable changes in size inequality following thinning due to the fact that most trees harvested during management were smaller (Figure 2), which reduced the numerical disparity between large and small trees, resulting in a more uniform stand structure. However, as retained trees grow and resource efficiency rises, new competitive relationships intensify, and the Gini coefficient rebounds with stand density. This implies that the complexity of stand structure is not permanently suppressed by thinning. Dominant tree species gradually reestablish growth advantages through canopy stratification and root system expansion (Forrester 2019), leading to a shift in stand productivity from being initially driven by smaller trees (immediately post‐thinning) to larger trees (Britwum et al. 2022). The rebound of the Gini was smaller in CNFM stands, suggesting that natural regeneration and multi‐tree mixing can effectively mitigate the extremes of tree size differences by promoting resource complementarity among different tree species. In contrast, in SBFM stands, the excessive pursuit of neat stand structure may exacerbate the cyclical fluctuations of the Gini, reflecting the instability of dominance among trees.

In newly established stands or those that have experienced intense harvesting, the growth rates of large and small trees tend to be similar. However, as the forest becomes denser over time, the gap in growth rates between large and small trees increases (Looney et al. 2018). In this experiment, the SGR and GDC change patterns in all managed stands were generally consistent throughout the survey period and remained below the levels observed in unmanaged stands. After management practices are implemented, the availability of light increased and soil nutrients were redistributed, allowing the remaining trees to access more light, nutrients, and growth space. However, the SGR and GDC decreased after an initial increase of 1–2 years, leading to a pronounced inverse asymmetry in SGR (Binkley et al. 2006), showing smaller trees consistently showed a growth advantage over larger ones during the survey period, which may be attributed to smaller trees benefiting more from releasing resources. Despite many small trees being harvested during management, their proportion remained dominant within the stand (Figure 2).

### 4.2 Dynamic Changes in BAI and Carbon Storage

Reduced stand density had a significant effect on carbon stocks at both the individual plant and stand levels. Management practices significantly increased tree‐level BAI, which is consistent with previous findings (Erkan et al. 2023), suggesting that resource release after thinning accelerated the growth response of the remaining trees. However, the effects of thinning on tree growth were not immediate but became increasingly evident as the forest progressed. Initial BAI growth in the managed stand was modest in years 1–2 after interseeding, but a significant growth acceleration occurred in years 3–5. This phenomenon can be attributed to adjustments in the pattern of resource competition, and the nonlinear relationship of time stand level BAI after management in this study also reflects the lag effect of management measures. In the early post‐interstory period, the remaining trees needed time to adapt to the altered light environment and competitive dynamics (Binkley et al. 2013). In subsequent years, canopy opening and soil nutrient redistribution combine to increase photosynthetic efficiency and carbon assimilation capacity (Pretzsch 2005).

Although the statistical analysis did not reveal a significant advantage of CNFM in carbon sequestration rates, the BAI model showed a more pronounced upward trend in stand‐level BAI in CNFM stands over time (Table 5), a finding that leads us to be cautiously optimistic about the long‐term performance of CNFM. We have highlighted the exploratory implications of these findings in Section 4. Unlike SBFM, which prioritizes structural regulation of target tree species, CNFM enhances stand species diversity by retaining economically valuable species and promoting seed dispersal (Ming et al. 2020). Nevertheless, the sustainability of this short‐term benefit requires long‐term validation. For instance, as residual trees increase in diameter, their resource demands may exceed environmental carrying capacity, potentially shifting growth dominance back to larger trees (Looney et al. 2018).

### 4.3 The Choice of Management Methods

From a managerial perspective, CNFM demonstrates greater short‐term applicability in high‐density, low‐quality *Pinus tabuliformis* plantations. Its “minimal intervention” principle prioritizes the restoration of ecosystem self‐regulation. It improves ecosystem stability and resistance to disturbance by retaining high economic value companion tree species (Ming et al. 2020). However, the universal applicability of CNFM's advantages may depend on management objectives. For example, if the goal is to balance timber production with biodiversity conservation, SBFM's parameterized structural controls offer precise operational guidance (Hui et al. 2018), whereas CNFM's “near‐natural” approach may trade partial economic yields for ecological benefits. Thus, CNFM's low‐disturbance characteristics are more sustainable in regions prioritizing ecological restoration, while SBFM could serve as a complementary strategy in extreme scenarios such as severe soil degradation or pest outbreaks.

## 5 Conclusion

Preliminary studies showed that the stand structure and above‐ground carbon stock exhibit unique change patterns under close‐to‐nature forest management and structure‐based forest management. After thinning, the Gini of the stands experienced a significant decline. However, Gini has been on the rise as competition between trees has resumed, which indicates that the complexity of the forest structure gradually restores. Growth partitioning analyses showed that larger trees contributed more to stand growth in unmanaged stands, while managed stands shifted growth dominance to smaller trees after treatment. Over time, larger trees gradually regained growth dominance, though full recovery required extended observation. Structure‐based forest management exhibited stronger fluctuations in growth dominance than close‐to‐nature management. Additionally, the carbon storage in the above‐ground of the managed stands remains below that of the unmanaged stands for a considerable period, and the carbon neutrality of close‐to‐nature management stands is projected to be achieved in 7.8 years, whereas structure‐based forest management stands require a marginally longer recovery period of 8.7 years to reach the net‐zero threshold.

Our study focused on evaluating the recovery effectiveness of plantations under different management methods, with the aim of providing important theoretical foundations for the management of *Pinus tabuliformis* plantations on the Loess Plateau. Regardless of the management model, it is conducive to the formation of a sound forest structure. If the management goal is to produce timber in a relatively short period, for *Pinus tabuliformis* plantations with high density and poor quality, close‐to‐nature management is recommended, exploiting their natural regeneration potential. While initial data suggest faster carbon recovery in close‐to‐nature management stands, the 5‐year observation period precludes definitive conclusions about long‐term superiority. Structure‐based forest management stands may exhibit delayed carbon compensation effects requiring extended monitoring. As small trees grow, the proportion of larger trees is expected to increase. Understanding when these larger trees will regain a growth advantage will be a key focus of future research.

## Author Contributions

**Jie Zhang:** investigation (equal), methodology (equal), visualization (equal), writing – original draft (lead), writing – review and editing (equal). **Huiyuan Yang:** investigation (equal), visualization (equal), writing – review and editing (equal). **Youding Zeng:** investigation (equal), visualization (equal), writing – review and editing (equal). **Sakib Al Hassan:** data curation (equal), writing – review and editing (equal). **Mahmuda Akter Jui:** data curation (equal), writing – review and editing (equal). **Li Gu:** conceptualization (equal), investigation (equal), writing – review and editing (equal).

## Conflicts of Interest

The authors declare no conflicts of interest.

## Data Availability

All data and code supporting the findings of this study have been permanently archived in the FigShare repository under a CC‐BY 4.0 license (https://doi.org/10.6084/m9.figshare.28219634).
